# Clinical outcomes in COVID-19 among patients with hypertension in the Philippine CORONA Study

**DOI:** 10.1186/s40001-022-00969-5

**Published:** 2023-02-03

**Authors:** Adrian I. Espiritu, Ma. Sergia Fatima P. Sucaldito, Deborah Ignacia D. Ona, Almira Doreen Abigail O. Apor, Marie Charmaine C. Sy, Veeda Michelle M. Anlacan, Roland Dominic G. Jamora

**Affiliations:** 1 Division of Adult Neurology, Department of Neurosciences, College of Medicine and Philippine General Hospital, University of the Philippines Manila, Manila, Philippines; 2 Department of Clinical Epidemiology, College of Medicine, University of the Philippines Manila, Manila, Philippines; 3 Department of Medicine (Division of Neurology) and Department of Psychiatry, University of Toronto, Toronto, ON Canada; 4 Department of Medicine, Philippine General Hospital, University of the Philippines, Manila, Philippines; 5 Division of Hypertension, Department of Medicine, Philippine General Hospital, University of the Philippines, Manila, Philippines; 6 Institute for Neurosciences, St. Luke’s Medical Center, Quezon City, Philippines; 7 Institute for Neurosciences, St. Luke’s Medical Center, Global City, Philippines

**Keywords:** COVID-19, SARS-CoV-2, Hypertension, Clinical outcomes, Mortality, Respiratory failure, ICU Admission, Retrospective cohort

## Abstract

**Objective:**

To describe the association between hypertension and clinical outcomes in a cohort of patients with coronavirus disease 2019 (COVID-19).

**Design:**

Retrospective cohort study.

**Setting:**

Thirty-seven (37) hospitals in the Philippines.

**Patients:**

10,881 patients admitted for COVID-19 from February to December 2020.

**Measurements and main results:**

Among the 10,881 patients included in the Philippine CORONA Study, 3647 (33.5%) had hypertension. On regression analysis adjusted for confounders (age group, sex, smoking history, diabetes, chronic cardiac disease, chronic kidney disease, chronic respiratory disease, chronic neurologic disease, chronic liver disease, HIV/AIDS, and malignancy), patients with hypertension had significantly greater odds of in-hospital mortality (OR 1.33, 95% CI 1.17–1.52), respiratory failure (OR 1.99, 95% CI 1.75–2.28), ICU admission (OR 2.16, 95% CI 1.90–2.45) and severe/critical disease (OR 1.57, 95% CI 1.41–1.75), compared to patients without hypertension. The time-to-event analysis with confounder adjustment also showed that hypertension was significantly associated with shorter time-to-event outcomes of in-hospital mortality (HR 1.13, 95% CI 1.01–1.26), respiratory failure (HR 1.86, 95% CI 1.65–2.10), and ICU admission (HR 1.99, 95% CI 1.76–2.23).

**Conclusions:**

Our analysis of nationwide data confirmed previous findings that hypertension is an independent risk factor for worse clinical outcomes among patients hospitalized for COVID-19, with increased odds of in-hospital mortality, respiratory failure, ICU admission, and severe/critical COVID-19. More specific studies should be done to elucidate the impact of hypertension characteristics, such as chronicity, severity, drug therapy, and level of control on these clinical outcomes.

## Introduction

Since December 2019, a novel coronavirus SARS-CoV-2 has swept all across the world. Globally, it has infected 500 million individuals, leading to more than 6 million deaths [1]. In the Philippines, more than 4 million people have been infected, with 64 thousand individuals expiring from the disease [2]. The COVID-19 pandemic continues to strain the healthcare system in terms of cost, resources, and workforce. Because of its impact on public health, there is an ongoing need to elucidate the pathophysiology of COVID-19 and the risk factors that may impact its transmission, virulence, and associated clinical outcomes. COVID-19 commonly presents with fever, cough, dyspnea, fatigue, headache [3], and disturbances of olfactory and gustatory function [4]. Rarely, it may also present with vasculitis-like skin lesions [5].

Early studies among patients with COVID-19 have identified hypertension as the most common comorbidity, suggesting that it may be an independent risk factor for increased severity and mortality among patients with COVID-19 [6, 7]. According to the latest National Nutrition Survey done by the Food and Nutrition Research Institute (FNRI) in 2018, among Filipinos 20–59 years of age, the prevalence of hypertension was 19.2%. Among adults aged 60 years and above, it was pegged at 35% [8]. These hypertensive individuals, constituting a sizeable bulk of the Filipino population, may be at higher risk for COVID-19 infection and disease progression.

Despite this, many earlier studies did not account for the confounding effect of comorbidities, such as diabetes mellitus, obesity, and coronary artery disease, that often cluster around hypertension. Succeeding studies since have shown heterogeneous results. Some studies showed that hypertension was an independent predictor of severity and mortality [9, 10]. Others discovered that it was only a predictor when combined with another comorbidity [11], while some investigators surmised that it was not a predictor at all [12–14]. There is still a continuing controversy on the effect of hypertension on the COVID-19 disease process. To help address this knowledge gap and contribute to the growing fund of knowledge on COVID-19, we performed an analysis of data from the Philippine COVID-19 outcomes: a retrospective study of neurological manifestations and associated symptoms (Philippine CORONA Study) [15] to elucidate the association between hypertension and clinical outcomes among Filipino patients hospitalized for COVID-19.

## Materials and methods

### Study design and source population

The Philippine CORONA Study was a multi-center retrospective cohort study that described the neurologic characteristics and clinical outcomes of patients hospitalized for COVID-19. It involved patients admitted to 37 participating hospitals in the Philippines from February to December 2020 [15]. The study protocol was reviewed and approved by the research ethics boards of participating sites and was registered with ClinicalTrials.gov (NCT04386083) [16]. To meet the objective of our study, we gathered hypertension data from the Philippine CORONA Study cohort and analyzed the association between hypertension and relevant clinical outcomes, such as COVID-19 disease severity, neurological outcomes, respiratory failure, dependence on mechanical ventilator (MV) > 5 days, need for ICU admission, prolonged ICU stay > 7 days, prolonged length of hospital stay > 14 days, and mortality.

### Exposure

The cohort of 10,881 patients was divided into groups with and without hypertension. The diagnosis of hypertension was determined by clinicians through past medical history and/or a physical examination finding of blood pressure greater than or equal to 140/90 during admission. Thus, the group with hypertension included both individuals with a prior diagnosis of hypertension and those patients who were newly diagnosed upon admission. No data were available on the control of hypertension or anti-hypertensive medications started prior to or during the admission.

### Outcomes

The primary outcomes of interest were in-hospital mortality, respiratory failure, and ICU admission. Secondary outcomes examined included COVID-19 disease severity at nadir (mild/moderate versus severe/critical COVID-19), neurological outcome (no improvement in neurologic symptoms versus partial or complete resolution of neurologic symptoms), prolonged mechanical ventilator (MV) dependence (defined as > 5 days on MV), prolonged ICU stay (defined as > 7 days), and prolonged hospital stay (defined as > 14 days).

### Statistical analysis

Baseline patient characteristics and outcomes were summarized using descriptive statistics. Data were assessed by the Shapiro–Wilk test to evaluate normality. Normally distributed continuous variables were described with means and standard deviations. Medians and interquartile ranges (IQR) were used to describe continuous variables that were not normally distributed. Categorical variables were described using counts and proportions. Baseline characteristics and clinical outcomes were compared between groups with and without hypertension. Significant differences in these groups were determined by Student’s *t* test for normally distributed continuous data, and Mann–Whitney *U* test for non-normally distributed variables. For categorical variables, heterogeneity of the proportions between the two groups was determined by the Chi-square test.

The associations between hypertension and the dichotomous outcomes of interest were determined by multivariable binary logistic regression. Survival analysis was also done for time-to-event data on mortality, respiratory failure, and admission to ICU. The time-to-event data were right-censored, using time-to-discharge as the exit from the analysis among those who did not experience the event of interest (e.g., mortality, respiratory failure, admission to ICU) during the hospital stay. The associations between hypertension and the different time-to-event outcomes of interest were determined by univariate Cox proportional hazards regression analysis. The logistic and Cox proportional hazards regression models used were adjusted for the following predetermined confounders: age group, sex, smoking status, diabetes, chronic cardiac disease, chronic respiratory disease, chronic kidney disease, chronic neurologic disease, chronic liver disease, HIV/AIDS, and malignancy. Kaplan–Meier curves adjusted to the different confounding variables of interest were constructed to compare the time-to-event curves of the groups with and without hypertension.

All statistical tests were two-tailed, and *p* < 0.05 was set as the threshold for statistical significance. All analyses were carried out in Stata Version 15.1 (StataCorp LLC, TX, USA).

## Results

### Baseline characteristics of analytic cohort

Among the 10,881 patients included in the Philippine CORONA Study, 3647 (33.5%) had hypertension (see Table 1). The median age of patients was 54 years (IQR 28 years), with 64.8% of patients aged less than 60 years. Patients in the hypertension group were more likely to be 60 years of age or more (55.6%, *p* < 0.001) and ever-smokers (15.6%, *p* < 0.001), but less likely to be female (46.9%, *p* < 0.001). Several comorbidities were significantly more common among the group with hypertension, such as diabetes mellitus (45.1%, *p* < 0.001), chronic cardiac disease (11.3%, *p* < 0.001), chronic respiratory disease (8.61%, *p* < 0.001), chronic kidney disease (13.0%, *p* < 0.001), chronic liver disease (0.80%, *p* = 0.015), and malignancy (0.08%, *p* < 0.001). There were significantly fewer patients with HIV/AIDS (*p* = 0.001) among the hypertensive group. Among neurologic comorbidities, cerebrovascular disease (7.95%, *p* < 0.001), neurodegenerative disease (0.93%, *p* < 0.001), and peripheral nerve and muscular disease (0.25%, *p* = 0.03) were more common among the hypertensive group.

**Table 1:** Baseline characteristics of patients in groups with and without hypertension

Features	All patients	Hypertensive	Non-hypertensive	*p*-value
	(*n* = 10,881)	(*n* = 3647)	(*n* = 7234)	
Socio-demographic data				
Age group				< 0.001
19–59 y, *n* (%)	7047 (64.8%)	1619 (44.4%)	5428 (75.0%)	
≥ 60 y, *n* (%)	3834 (35.2%)	2028 (55.6%)	1806 (25.0%)	
Female, *n* (%)	5099 (46.9%)	1591 (43.6%)	3508 (48.5%)	< 0.001
Ever-smoker (past/current), *n* (%)	1026 (9.4%)	570 (15.6%)	456 (6.3%)	< 0.001
Non-neurologic comorbidities, *n* (%)				
Diabetes mellitus	2191 (20.1%)	1643 (45.1%)	548 (7.6%)	< 0.001
Chronic cardiac disease^*a*^	512 (4.7%)	411 (11.3%)	101 (1.4%)	< 0.001
Chronic respiratory disease^*b*^	616 (5.7%)	314 (8.6%)	302 (4.2%)	< 0.001
Chronic kidney disease	611 (5.6%)	474 (13.0%)	137 (1.9%)	< 0.001
Chronic liver disease	60 (0.6%)	29 (0.8%)	31 (0.4%)	0.015
HIV/AIDS	37 (0.3%)	3 (0.1%)	34 (0.5%)	0.001
Malignancy	244 (2.2%)	113 (3.1%)	131 (1.8%)	< 0.001
Past neurologic history, *n* (%)				
Stroke/cerebrovascular	321 (3.0%)	290 (8.0%)	31 (0.4%)	< 0.001
Epilepsy	27 (0.3%)	13 (0.4%)	14 (0.2%)	0.107
Neurodegenerative^*c*^	44 (0.4%)	34 (0.9%)	10 (0.1%)	< 0.001
Headache syndrome	5 (0.1%)	1 (0.03%)	4 (0.1%)	0.670
Demyelinating disorder	2 (0.02%)	2 (0.02%)	–	0.112
Central nervous system (CNS) infection	5 (0.1%)	1 (0.03%)	4 (0.1%)	0.670
Peripheral nervous system (PNS) disorders^*d*^	15 (0.1%)	9 (0.3%)	6 (0.1%)	0.030
Respiratory and constitutional symptoms, *n* (%)				
Fever	3927 (36.1%)	2034 (55.8%)	1893 (26.2%)	< 0.001
Cough	4411 (40.5%)	2317 (63.5%)	2094 (29.0%)	< 0.001
Dyspnea	2703 (24.8%)	1534 (42.1%)	1169 (16.2%)	< 0.001
Rhinorrhea	607 (5.6%)	231 (6.3%)	376 (5.2%)	0.015
Sputum production	637 (5.9%)	336 (10.0%)	271 (3.8%)	< 0.001
Sore throat	751 (6.9%)	294 (8.1%)	457 (6.3%)	0.001
Diarrhea	597 (5.5%)	282 (7.7%)	315 (4.4%)	< 0.001
Fatigue	713 (6.6%)	382 (10.5%)	331 (4.6%)	< 0.001
Others	1674 (15.4%)	601 (16.5%)	1073 (14.8%)	0.025
New-onset neurological symptoms, *n* (%)				
Headache	607 (5.6%)	220 (6.0%)	387 (5.4%)	0.143
Nausea or vomiting	158 (1.5%)	86 (2.4%)	72 (1.0%)	< 0.001
Seizure	96 (0.9%)	57 (1.6%)	39 (0.5%)	< 0.001
Altered mental state^*e*^	518 (4.8%)	314 (8.6%)	204 (2.8%)	< 0.001
Olfactory or taste dysfunction	663 (6.1%)	250 (6.6%)	413 (5.7%)	0.018
Dysfunctions of other senses^*f*^	166 (1.5%)	93 (2.6%)	73 (1.0%)	< 0.001
Bulbar symptoms^*g*^	122 (1.1%)	91 (2.5%)	31 (0.4%)	< 0.001
Motor symptoms	246 (2.3%)	174 (4.8%)	72 (1.0%)	< 0.001
Sensory symptoms	53 (0.5%)	37 (1.0%)	16 (0.2%)	< 0.001
Myalgia	256 (2.4%)	87 (2.4%)	169 (2.3%)	0.873
Others^*h*^	33 (0.3%)	25 (0.7%)	8 (0.1%)	< 0.001
New-onset neurological disorders/complications, *n* (%)				
Encephalopathy^*i*^	644 (5.9%)	396 (10.9%)	248 (3.4%)	< 0.001
Symptomatic seizure/status epilepticus	125 (1.1%)	75 (2.1%)	50 (0.7%)	< 0.001
Stroke/cerebrovascular^*i*^	367 (3.3%)	255 (7.0%)	112 (1.6%)	< 0.001
CNS infection^*k*^	7 (0.1%)	1 (0.03%)	6 (0.1%)	0.436
Others^*l*^	14 (0.1%)	5 (0.1%)	9 (0.1%)	1.000
Treatment/s received, *n* (%)				
Glucocorticoids	2844 (26.1%)	1717 (47.1%)	1127 (15.6%)	< 0.001
Tocilizumab	1029 (9.5%)	672 (18.4%)	357 (4.9%)	< 0.001
Antiviral^*m*^	1902 (17.5%)	1178 (32.3%)	724 (10.0%)	< 0.001
Antibacterial	9014 (82.8%)	3339 (91.6%)	5675 (78.5%)	0.001
Others^*n*^	3905 (35.9%)	1601 (43.9%)	2304 (31.9%)	< 0.001

^a^Includes heart failure, coronary artery disease, prior history of myocardial infarction, and other cardiac conditions

^b^Includes bronchial asthma, chronic obstructive pulmonary disease, restrictive lung disease, and other pulmonary conditions

^c^Includes dementia, and movement disorders

^d^Includes PNS infection, peripheral nerve disease, neuromuscular junction disorder, and muscle disorder

^e^Includes altered sensorium, and confusion

^f^Includes visual, hearing, and vestibular dysfunctions

^g^Includes facial paresthesia, facial weakness, dysarthria, dysphonia, dysphagia, tongue weakness, and neck weakness

^h^Includes tremor, dystonia, choreoathetosis, bradykinesia, ataxia, and meningismus

^i^Includes encephalopathy, and anoxic brain injury

^j^Any acute cerebrovascular disease (CVD) (no need to distinguish between CVD infarction, hemorrhage)

^k^Includes encephalitis, meningitis, and meningoencephalitis

^l^Includes acute disseminated encephalomyelitis, optic neuritis, sensory ganglionitis, radiculitis, anterior horn syndrome, peripheral neuritis [Guillain Barre Syndrome (GBS), other than GBS], neuromuscular disorder, and myositis

^m^Includes remdesivir, lopinavir, and ritonavir

^n^Includes chloroquine, hydroxychloroquine, convalescent plasma, and other therapies

Baseline data also showed that hypertensive patients who were admitted for COVID-19 were more likely to be symptomatic than their non-hypertensive counterparts. Symptoms such as fever (55.8%, *p* < 0.001), cough (63.5%, *p* < 0.001), dyspnea (42.1%, *p* < 0.001), rhinorrhea (6.33%, *p* = 0.015), sputum production (10.0%, p < 0.001), sore throat (8.06%, *p* = 0.001), diarrhea (7.73%, *p* < 0.001), and fatigue (10.5%, *p* < 0.001) were all more common in the hypertensives. Patients with hypertension also more often presented with nausea and vomiting (2.36%, *p* < 0.001), seizure (1.56%, *p* < 0.001), altered mental state (8.61%, *p* < 0.001), olfactory and taste dysfunction (6.58%, *p* = 0.018), motor (4.77%), and bulbar (2.50%), and sensory (1.01%) symptoms (*p* < 0.001). Neurologic diagnoses of encephalopathy (10.9%, *p* < 0.001), status epilepticus (2.06%, *p* < 0.001), and stroke (6.99%, *p* < 0.001) were also more often made among the hypertensive group compared to the non-hypertensive group. Regarding treatments received, a significantly more significant proportion of patients in the hypertension group were given glucocorticoids (47.1%, *p* < 0.001), tocilizumab (18.4%, *p* < 0.001), antiviral (e.g., remdesivir, lopinavir/ritonavir) (32.3%, *p* < 0.001), and antibiotics (91.6%, *p* = 0.001) than their non-hypertensive counterparts.

### Outcomes

A total of 4061 patients (37.3%) were classified as severe or critical cases of COVID-19 at nadir and 1702 patients (15.6%) expired during admission (see Table 2). The group with hypertension had a significantly higher percentage of severe/critical cases (52.4%) than the group without hypertension (30.4%). There was also a significantly greater mortality rate in the group with hypertension (23.96%) than without (11.45%), with a median time-to-mortality of 16 days for hypertensives and 14 days for non-hypertensives.

**Table 2:** Clinical outcomes of COVID-19 patients with and without hypertension

Outcomes	Hypertensive	Non-hypertensive	p-value
	(*n* = 3647)	(*n* = 7234)	
COVID-19 severity at nadir			< 0.001
Mild, *n* (%)	1714 (47.6%)	4976 (69.6%)	
Severe/critical, *n* (%)	1888 (52.4%)	2173 (30.4%)	
In-hospital mortality	874 (24.0%)	828 (11.5%)	< 0.001
Time to in-hospital mortality in days, median (IQR)	16 (14)	14 (12)	< 0.001
Respiratory failure, *n* (%)	1005 (27.6%)	603 (8.3%)	< 0.001
Time to respiratory failure in days, median (IQR)	5 (4)	5 (4)	0.896
Duration of MV in days, median (IQR)	13 (13)	12 (11)	0.9136
MV dependence ≤ 5 days, n (%)	131 (13.0%)	87 (14.5%)	0.4715
MV dependence > 5 days, n (%)	873 (87.0%)	515 (85.5%)	
Admitted to ICU, *n* (%)	1122 (30.8%)	618 (8.5%)	< 0.001
Time to ICU admission in days, median (IQR)	5 (4)	4 (4)	0.660
Length of ICU stay in days, median (IQR)	15 (13)	15 (11)	0.885
ICU stay ≤ 7 days, *n* (%)	172 (15.3%)	100 (16.2%)	0.640
ICU stay > 7 days, *n* (%)	950 (84.7%)	518 (83.8%)	
Length of hospital stay^*a*^ in days, median (IQR)	14 (10)	13 (8)	< 0.001
Hospital stay ≤ 14 days, *n* (%)	2058 (56.4%)	4519 (62.5%)	< 0.001
Hospital stay > 14 days, *n* (%)	1589 (43.6%)	2715 (37.5%)	
Neurologic presentation or complication, *n* (%)	1084 (29.7%)	1207 (16.7%)	< 0.001
Neurologic outcome^*b*^			< 0.001
Full/partial neurologic recovery, *n* (%)	700 (81.8%)	939 (89.5%)	
No recovery, *n* (%)	156 (18.2%)	110 (10.5%)	

*MV* mechanical ventilation; *COVDI-19 *coronavirus disease 2019; *ICU* intensive care unit;* IQR* interquartile range

^a^Derived from overall length of stay for patients who were never admitted to ICU; excludes length of ICU stay for those who were admitted in the ICU

^b^Patients with recorded data for neurologic outcome (*n* = 1905)

Overall, 1608 patients (14.8%) developed respiratory failure requiring MV and 1740 patients (16.0%) required admission to the ICU. There was a significantly greater proportion of hypertensives who developed respiratory failure (27.6%, *p* < 0.001) and who were admitted to the ICU (30.8%, *p* < 0.001), but there was no significant difference between the groups in terms of time-to-respiratory failure, prolonged MV dependence (> 5 days), time-to-ICU admission, or prolonged ICU stay (> 7 days). More hypertensive patients had a prolonged hospital stay (> 14 days) (*p* < 0.001). There was a negative association between hypertension and partial or full neurologic recovery (*p* < 0.001).

### Logistic regression analysis

Logistic regression analysis of hypertension with the outcomes of interest was performed and adjusted for predetermined confounding variables, including age group, sex, smoking history, diabetes mellitus, chronic cardiac disease, chronic kidney disease, chronic respiratory disease, chronic neurologic disease, chronic liver disease, HIV/AIDS, and malignancy (see Table 3). Hypertension was associated with greater odds of severe/critical COVID-19 at nadir (adjusted OR 1.57, [95% CI 1.41–1.75], *p* < 0.001), neurologic complications (adjusted OR 1.54, [95% CI 1.37–1.73], *p* < 0.001), in-hospital mortality (adjusted OR 1.33, [95% CI 1.17–1.52], *p* < 0.001), respiratory failure (adjusted OR 1.99, [95% CI 1.75–2.28], *p* < 0.001), and ICU admission (adjusted OR 2.16, [95% CI 1.90–2.45], *p* < 0.001). There was no sufficient evidence to suggest an association of hypertension with full/partial neurologic improvement, prolonged ICU stay > 7 days, or prolonged hospital stay > 14 days.

**Table 3:** Association of having hypertension with the different outcomes of interest

Regression analysis	Adj. OR^a^	95% CI	*p*-value
Severe/critical COVID-19 at nadir	1.57	1.41, 1.75	< 0.001
Neurological presentation/complication	1.54	1.37, 1.73	< 0.001
Full/partial neurological improvement	0.91	0.66, 1.24	0.530
In-hospital mortality	1.33	1.17, 1.52	< 0.001
Respiratory failure	1.99	1.75, 2.28	< 0.001
MV dependence > 5 days	0.49	0.36, 0.68	< 0.001
ICU admission	2.16	1.90, 2.45	< 0.001
ICU stay > 7 days	1.07	0.80, 1.42	0.667
Hospital stay > 14 days	1.06	0.96, 1.17	0.217
Time–to-event analysis	Adj. HR^b^	95% CI	p-value
In-hospital mortality	1.13	1.01, 1.26	0.038
Respiratory failure	1.86	1.65, 2.10	< 0.001
ICU admission	1.99	1.76, 2.23	< 0.001

*MV* mechanical ventilation; *ICU* intensive care unit; *COVID-19* coronavirus disease 2019

^a^Individual univariate multiple logistic regression analysis with independent variable hypertension adjusted for age group, sex, smoking history, diabetes, chronic cardiac disease, chronic kidney disease, chronic respiratory disease, chronic neurologic disease, chronic liver disease, HIV/AIDS, and malignancy

^b^Individual univariate multiple Cox proportional hazards regression analysis with independent variable hypertension adjusted for age group, sex, smoking history, diabetes, chronic cardiac disease, chronic kidney disease, chronic respiratory disease, chronic neurologic disease, chronic liver disease, HIV/AIDS, and malignancy.

### Time-to-event analysis

**Fig. 1:**
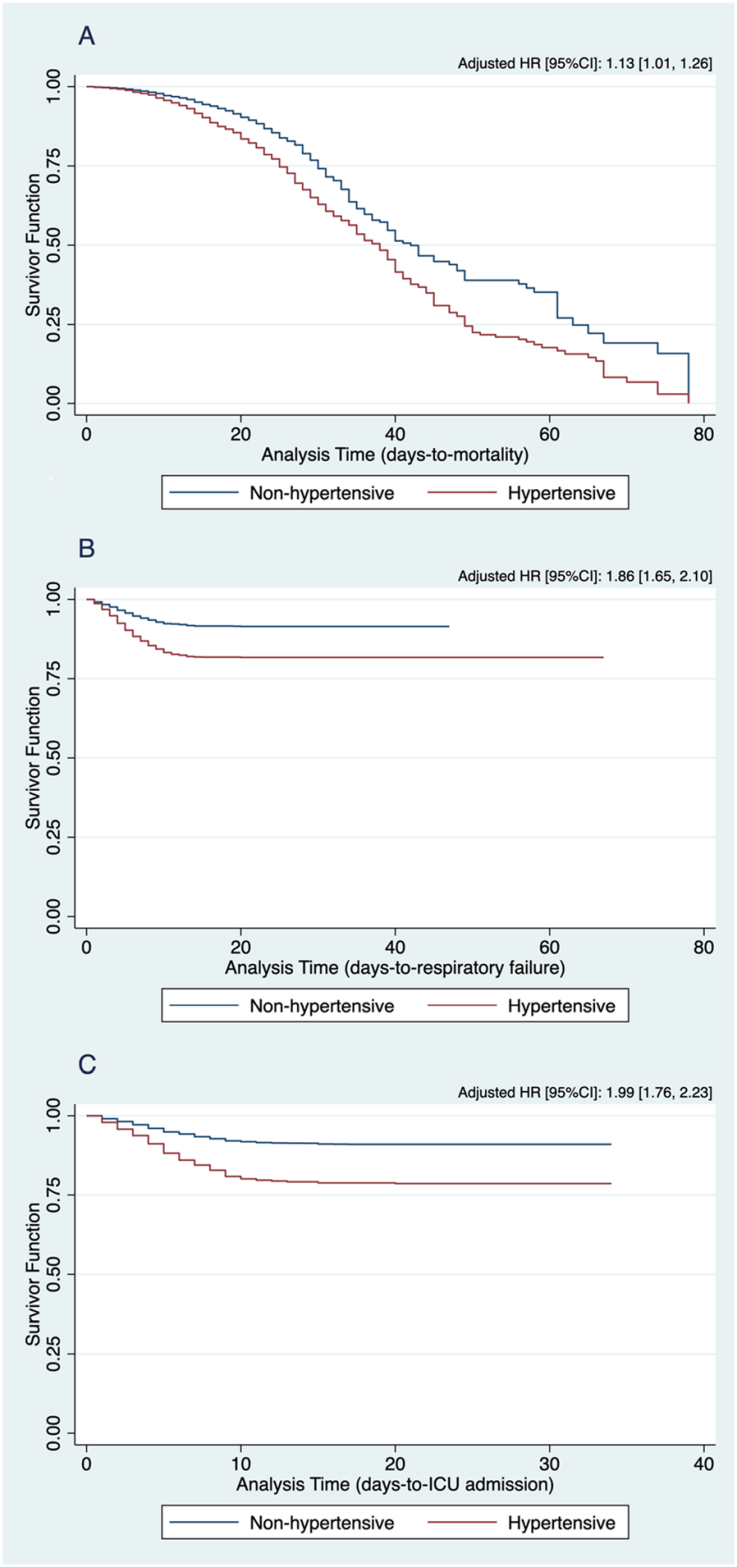
Comparison of Kaplan–Meier curves for in-hospital mortality (**A**), respiratory failure (**B**), and ICU admission (**C**) between hypertensive and non-hypertensive COVID-19 patients, adjusted for the different confounding variables of interest

Univariate multiple Cox proportional hazards regression analysis was done to determine the association of hypertension with time-to-event of clinical outcomes, including in-hospital mortality, respiratory failure, and ICU admission (see Fig. 1). After adjusting for predetermined confounding variables, hypertension was significantly associated with shorter time-to-event outcomes of in-hospital mortality (HR 1.13, [95% CI 1.01–1.26], *p* = 0.038), respiratory failure (HR 1.86, [95% CI 1.65–2.10], *p* < 0.001), and ICU admission (HR 1.99, [95% CI 1.76–2.23], *p* < 0.001).

## Discussion

The Philippine CORONA Study data paved the way for sub-studies that elucidated the impact of comorbidities on the outcomes of COVID-19, including studies on body mass index [17], diabetes mellitus [18], malignancy [19], and stroke [20]. Our findings from the Philippine CORONA Study data were consistent with the findings of past observational studies [6, 7, 9, 10] showing that hypertension is an independent risk factor for worse clinical outcomes among patients hospitalized for COVID-19. In our analysis, hypertension was shown to have a significant association with in-hospital mortality, respiratory failure, ICU admission, severe/critical COVID-19 at nadir, and neurologic complications.

Since it is the most common comorbidity among patients with COVID-19, several studies were done to determine the relationship between hypertension with poor clinical outcomes in this patient population. These studies were heterogeneous in their findings, with some showing a positive association [9, 10], while others showed no association with outcomes such as mortality or severity of COVID-19 [12–14]. Due to its complexity, the impact of hypertension on the COVID-19 disease course (and vice-versa) has been difficult to characterize. An early study on hospitalized patients suggested that elevated systolic blood pressure and blood pressure variability were associated with higher mortality, ICU admission, and COVID-induced heart failure [21]. Another observational study showed a similar association but found no significant difference in outcomes among the different grades of hypertension (i.e., grade 1 hypertension versus grade 2 or 3 hypertension) [22]. Further study needs to be done among the hypertensive population to determine if the severity of hypertension and level of control has any effect on COVID-19 clinical outcomes. Data are also scarce on comparisons of outcomes between hypertensive patients who are chronically hypertensive versus newly diagnosed.

The pathophysiologic mechanisms to explain the poorer outcomes observed in hypertensive patients with COVID-19 are also under investigation. It is suggested that chronically hypertensive patients have greater endothelial dysfunction and hypertension-mediated organ damage, increasing their susceptibility to cardiovascular complications if they are infected with COVID-19 [23]. It is also hypothesized that hypertension and SARS-CoV-2 interact with the ACE/Angiotensin II/AT1R axis, which promotes vasoconstriction and RAAS upregulation, as well as the vasodilatory ACE2/Ang (1–7)/AT2R axis, to promote viral entry, replication, and organ damage [24, 25]. Recent studies on hypertension and atherosclerosis have shown that immune cell infiltration and cytokine production play a role in sustaining elevated blood pressure and target organ damage [26]. As such, the pro-inflammatory cascade brought about by COVID-19 infection may compound the existing chronic inflammation in hypertensive patients. Supporting this hypothesis, findings of a study also demonstrated that the immune cells in the airways of COVID-19 patients with hypertension exhibited inflammatory signals, which correlated with COVID-19 disease progression [27]. To date, no studies have yet specifically explored the association of hypertension with COVID-19-related cytokine storm.

Hypertension is a well-known and prevalent risk factor for cardiovascular disease. Hypertension occurs in conjunction with several modifiable and non-modifiable factors that often cluster and work in synergy, such as in metabolic syndrome. We confirmed this clustering of comorbidities in our analysis of the Philippine CORONA Study data. Earlier studies that showed an association of hypertension with poor clinical outcomes did not consider these comorbidities, which are likely to confound the observations. In one study, hypertension alone did not affect mortality or ARDS in COVID-19, but there was an association if considered together with diabetes [11]. In another study, neither hypertension nor diabetes mellitus affected the clinical outcomes in critically ill COVID-19 patients [12]. We adjusted our regression and time-to-event analyses for these predetermined confounders and found that the positive association of hypertension with poor outcomes remains significant.

The burden of hypertension and its interaction with COVID-19 is not only limited to the comorbid itself but also the medications used to treat the condition. In particular, animal studies have shown that renin–angiotensin–aldosterone system (RAAS) inhibitors increase the expression of ACE2 receptors, which constitute one of the initial steps in COVID-19 viral entry into cells [28]. These findings led to succeeding studies investigating the association of using these anti-hypertensive medications on clinical outcomes, such as worsening severity and mortality. Although initially thought to increase the risk of COVID-19 infection, using RAAS inhibitors did not significantly increase the risk of infection or mortality from COVID-19 in several observational studies [29–31]. A large study involving 16866 cases of COVID-19 in the United Kingdom showed that using RAAS blockers, calcium channel blockers, and thiazides among hypertensives were associated with a lower risk of infection and no effect on mortality [32]. More recently, a randomized clinical trial BRACE CORONA involving 659 patients hospitalized for COVID-19 showed that continuing RAAS inhibitors during COVID-19 hospitalization versus discontinuing them did not affect days alive and out-of-hospital in 30 days, mortality, cardiovascular death, or COVID-19 progression [33]. The European Society of Cardiology, Italian Society of Hypertension, and British Cardiovascular Society have also released their official statements on the safety of continuing RAAS inhibitors among patients who have conditions for which these are indicated [34–36]. There is also a hypothesis that beta-blockers, unlike RAAS blockers, may improve outcomes in COVID-19 patients, as they were found to reduce the expression of ACE2 receptors and interleukin-6 [37]. In the UK study, beta-blocker use was even initially associated with higher odds of COVID-19 infection, but this effect was attenuated after adjusting for confounders. Succeeding studies on the association of different classes of anti-hypertensives with outcomes discovered no significant impact on the risk of COVID-19 infection, need for MV, and mortality [9, 38, 39].

Currently, several therapeutics have been shown to prevent disease progression among patients with mild and moderate diseases. In phase 3 trials, antivirals nirmatrelvir–ritonavir [40] and molnupiravir [41] were shown to reduce the composite risk of hospitalization and 28-day mortality among symptomatic, unvaccinated adults with at least one risk factor for progression. While the nirmatrelvir–ritonavir trial included hypertension as a risk factor for progression, the molnupiravir trial did not. Our findings serve to strengthen the role of hypertension as a risk factor for COVID-19 disease progression and promote its consideration as an additional indication for prescribing these promising therapeutics.

In the Philippines, a sizeable proportion of the population has hypertension, ranging from 19.2% among adults 20–59 years of age to 35% for those aged 60 years and above [8]. Based on previous studies and confirmed by our findings, individuals with hypertension are at significantly higher risk of mortality and poor clinical outcomes once infected with COVID-19. Since they are at risk for disease progression requiring mechanical ventilation and intensive care, hypertensive patients with COVID-19 represent a vulnerable population. Further study must be done to determine which subsets of the hypertensive population, if any, are most at risk and if they would benefit from intensified protective measures, such as vaccine prioritization and antiviral distribution.

Our data were limited and did not include hypertension-related characteristics, such as chronicity, severity of hypertension, level of blood pressure control, and anti-hypertensives used. Data on whether hypertension was primary or secondary were also unavailable. These factors may affect the pathophysiology of COVID-19 through worsened atherosclerosis, endothelial dysfunction, and target organ damage such as myocardial injury.

## Conclusions

Our analysis of nationwide data confirmed previous findings that hypertension is an independent risk factor for worse clinical outcomes among patients hospitalized for COVID-19, with increased odds of in-hospital mortality, respiratory failure, ICU admission, severe/critical COVID-19 at nadir, and neurologic complications. More specific studies should be done to clarify the impact of hypertension characteristics, such as chronicity, severity, and level of control on these clinical outcomes.

## Data Availability

All data related to this research have been included in this paper.
